# Working memory training in children with developmental language disorder: Effects on complex syntax in narratives

**DOI:** 10.3389/fresc.2022.1068959

**Published:** 2023-01-04

**Authors:** Hélène Delage, Emily Stanford, Clara Baratti, Stéphanie Durrleman

**Affiliations:** ^1^ Équipe de Psycholinguistique et Logopédie, Faculté de Psychologie et des Sciences de L'Éducation, Université de Genève, Genève, Switzerland; ^2^ Department of English Language and Literature, Faculty of Humanities, University of Geneva, Geneva, Switzerland; ^3^ Department of Psychology, University of Milano-Bicocca, Milan, Italy; ^4^ Doctoral School in Humanities and Social Sciences, Faculty of Arts, Department of Languages, University of Helsinki, Helsinki, Finland; ^5^ Department of Linguistics, Faculty of Humanities, University of Geneva, Geneva, Switzerland; ^6^ Faculty of Science and Medicine, University of Fribourg, Fribourg, Switzerland

**Keywords:** developmental language disorder (DLD), syntax, narratives, working memory, training, children

## Abstract

This study assesses the impact of a working memory training program on the syntactic complexity of the spontaneous speech of French-speaking children with Developmental Language Disorder (DLD). Thirty-nine 6- to 12-year-old children with DLD were allocated to a WM training (DLD^MM^, *N* = 20) or an active control group (DLD^SQULA^, *N* = 19). The computerized training sessions took place three times a week, yielding 12 training hours per participant. Syntactic complexity was assessed in storytelling, measuring mean length of utterances, use of embedded clauses and rate of errors in complex utterances. The performance of participants with DLD was first compared to previous spontaneous data of 40 typically-developing (TD) children of the same age. Then, intragroup (pre- vs. post-test) and intergroup (DLD^MM^ vs. DLD^SQULA^) comparisons were made to assess the impact of the working memory training on the language measures. Global results confirmed syntactic impairment in children with DLD, as opposed to TD children, with large differences for the use of embedded clauses. Findings also suggested gains in the mastery of embedded clauses in children who participated in the WM training, whereas no gains were observed in the DLD control group. These findings confirm deficits in complex syntax in children with DLD, in particular in embedded clauses, and may encourage the clinical use of language sample analysis, which provides an ecological account of children's language performance. While our results should be replicated on a larger scale, they also suggest positive transfer effects of working memory training on the capacity of participants with DLD to produce embedded clauses, in line with previous studies showing a positive effect of WM training on tasks of expressive syntax. It thus seems that working memory training can yield benefits for language, which leaves open the door to new therapeutic approaches for children with DLD.

## Introduction

The present study investigates the relation between language and cognition, and more particularly between syntax and working memory (WM), an aspect of cognition that has been attracting attention in psycholinguistics (1, 2). Indeed, studies conducted with typically-developing (TD) children have shown connections between WM capacities and the mastery of syntactically complex sentences (3–6). This link between WM and syntactic complexity is of particular interest for language disorders, notably for children with Developmental Language Disorder (DLD). In this population, persistent grammatical impairment impacts their everyday life (7) and difficulties with complex syntax have been found to be linked to comorbid WM deficits (8–10). Inspired by these findings, our work investigates the effects of WM training on the syntax of French-speaking children with DLD. More specifically, the present study focuses on embedded clauses produced in spontaneous narrative samples, which is part of a larger-scale experiment dedicated to WM training and its impact on language of children with DLD. Promising results have already been obtained within this project for the production of accusative clitics (11) and of relative clauses (12).

### Syntax in DLD

According to the recent consensus (7, 13) and the DSM-5 (14), DLD is defined as a developmental and persistent disorder affecting oral language acquisition in children who do not present any signs of neurological damage, sensorial disabilities such as deafness, severe cognitive impairment, and/or pervasive developmental disorders. Language impairment in this condition is mainly apparent in phonology and morphosyntax (15, 16) and coexists with specific difficulties in word learning/semantics (17, 18). Aside from the core linguistic deficits in this condition, children with DLD also display numerous weaknesses in non-linguistic domains, such as deficits in auditory processing (19–21), motor disorders (22) or general executive impairments, including WM deficits (23).

**Figure 1:**
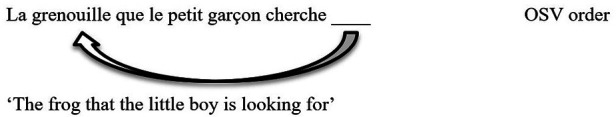
Object relative clause in French.

The syntactic difficulties attested in DLD affect complex syntactic structures, which include passive clauses (24–26), object relatives (27–29), object Wh-questions (30–32), as well as, for French-speaking children, accusative clitics (8, 33, 34). Delage and Frauenfelder (9) observe that these deficits all involve syntactic movement yielding non-canonical word order, i.e., with the object preceding the subject in an otherwise SVO (Subject-Verb-Object) language. This is illustrated for French in Figure 1 by an object relative clause.

Children with DLD are also known to experience difficulties with another type of structure that does not necessarily involve syntactic movement but is nonetheless syntactically complex: embedding (9, 35–37). Embedded clauses produced in spontaneous language by children and adolescents with DLD are indeed less frequent than attested in TD children, whether they are monolingual (9, 38) or bilingual (36), and they contain more errors (39). Hamann and colleagues (40) explored performance in spontaneous language samples of TD children aged 6, 8 and 11, compared to children (aged 6–10) and adolescents with DLD (aged 11–16). This study confirmed that the frequency of embedding, and of relative clauses in particular, was lower amongst individuals with DLD (both in children and adolescents) than that of TD children and contained a higher occurrence of ungrammatical utterances compared to TD children. As for older participants, i.e., adolescents with DLD aged 11 to 16, the authors emphasized the fact that the syntactic complexity of the utterances they produced was similar to that of younger children with DLD, aged 5 to 10, revealing the persistence of syntactic limitations with age.

Previous studies conducted on spontaneous language samples also highlighted the impact of syntactic complexity on children's performance in natural contexts. For instance, Gillam and Johnston (41) found that children with DLD, aged 9 to 12, produced more grammatical errors in complex sentences, defined by utterances containing two clauses, a main clause as well as another additional clause, than in simple sentences, consisting of a single (main) clause, compared to age-matched TD children in both spoken and written narratives. More recently, Marinellie's study (42) reported that children with DLD (mean age = 10;8) produced sentences that were significantly less complex, combined fewer complex sentence structures, and produced fewer clauses per utterance, than TD children of the same age in child-adult conversational language samples.

The low frequency of embedded clauses produced by children with DLD, as well as the high frequency of errors in their embedded clauses, has been explained in terms of the Derivational Complexity Hypothesis (DCH) (15, 30). According to the DCH, which follows the Minimalist Program's framework (43), the more complex a syntactic structure, the harder it is for children to produce it, giving rise to avoidance of syntactically complex structures. The complexity of syntactic structures is characterized by the nature and number of syntactic operations: On the one hand, the nature of the structures is complexified by syntactic movement or embedding, while on the other hand a larger amount of movement or embedding implies a higher degree of complexity. Thus, in this framework, the sentence in (a) below is more complex than the one in (b), because even though both involve embedding, the former includes more instances of embedding, represented by squared brackets.

(a) Mary knows [that John thinks [that the frog is in the garden]].
(b) John thinks [that the frog is in the garden]

Crucially for the current study, Jakubowicz (30) explains differences between children's and adults' syntax in terms of limited performance systems, such as working memory or attentional capacities [see also (44)^1^]. It is well known that executive functions gradually develop with age until reaching a performance similar to adults in typical development (46). It is also well established that executive functions interact with the language faculty (47–50) and seem to be strongly linked to syntactic processing [see (51) in preschoolers, or (52) in monolingual and bilingual school-aged children]. Consequently, young TD children, whose cognitive capacities are not yet fully developed, will produce fewer occurrences and more erroneous complex syntactic structures than older children and adults. In sum, the DCH framework accounts for syntactic disorders in individuals with DLD by an incomplete maturation of their executive functions, and in particular by persistent limitations in WM (9, 15).

### Working memory in DLD

WM deficits are well recognized in children with DLD [see (23) for a review]. WM is defined by Baddeley [(1), *p*. 189] as a system that « involves the temporary storage and manipulation of information that is assumed to be necessary for a wide range of complex cognitive activities », such as language comprehension and production. In Baddeley's tripartite model of WM^2^, three components play different roles: (1) a primary attentional control system, the *central executive*, rules over and connects two subsystems, namely (2) the *phonological loop* which stores acoustic and verbal information and (3) the *visuospatial sketchpad* which stores visuospatial information. Verbal simple-span tasks assess capacities of the phonological loop with tasks soliciting the simple storage and retrieval of verbal information such as repetition of digits, words, or non-words. In contrast, the central executive's evaluation is achieved with complex-span tasks that require maintaining information while manipulating information simultaneously.

Whereas capacities in simple and complex spans gradually improve in TD children and reach adult levels by adolescence (54), WM development of children with DLD is affected from an early age [see (55) in children aged 4–5] and does not normalize at adolescence and adulthood (56, 57). More precisely, it has been shown that (verbal) simple-span capacity of children with DLD is lower than that of TD children in measures of digit and word repetition (9, 58–60). Moreover, the persistent difficulties found in non-word repetition are considered clinical markers of DLD (61–65). WM deficits in DLD have also been repeatedly reported in complex-span tasks, such as backward digit span, listening span or counting span (9, 58, 59, 66–71). Such deficits were most often reported for verbal WM [as in 10 studies present in the review of Kapa and Plante (23)], but some studies also found group differences between TD and DLD for non-verbal WM [4 studies in Kapa and Plante (23)].

### Working memory and syntax

Several studies have demonstrated a close link between WM and syntax and, more specifically, a special relationship between WM capacities and complex syntax rather than syntax in general, whether for TD children (4, 6, 72) or for children with DLD (9, 10, 59, 73, 74)^3^. For example, Frizelle and Fletcher (10) have reported that WM scores significantly correlated with the production of relative clauses in a group of children with DLD aged 6 to 8. The participants of this study were asked to repeat complex sentences which consisted of sentences involving different types of embedded clauses with varying degrees of complexity. Results showed that, on the one hand, simple span scores, including word/non-word repetition and forward digit span, correlated with simple embedded clauses such as relative clauses that express a single proposition. On the other hand, complex span scores, including listening recall, counting recall, and backward digit span, strongly correlated with syntactically more complex embedded clauses such as biclausal relative constructions. In Delage and Frauenfelder's study (9), performance of participants with DLD aged from 5 to 14 was compared to that of TD participants of the same age in WM, with simple and complex-span tasks, and in complex syntax: production, repetition and comprehension of complex sentences. Results showed that age, simple and complex-span scores accounted for major parts of the variance (50%–58%) of scores in the complex sentence comprehension and repetition tasks, in both TD and DLD groups.

### Working memory training

Given results indicating that WM capacities predict performance in complex syntax in both TD and DLD groups, it seems logical to train the working memory of children who have deficits in this area, hoping for a positive transfer effect on their syntactic skills. Nevertheless, meta-analyses (76, 77) have reported that while WM training can improve WM capacities, the effects are usually limited to memory abilities without transfer to other cognitive abilities. However, it should be noted that the majority of studies included in these meta-analyses focused on participants with no particular disorders. As a result, it may have been difficult to improve skills because they were already functioning at an optimal level for the age of the participants. Moreover, while WM training has also been offered to participants with attention deficit hyperactivity disorder (78) or learning disorders (79), few studies have focused on children with DLD. One which did is Holmes and colleagues (80) who trained children with poor language skills aged 8 to 11 years by means of the *Cogmed* program (www.cogmed.com), a wide-ranging WM training program which includes a large number of visual WM tasks. The presence of visuospatial WM disorders is still a matter of debate in children with DLD (81–83), which contrasts with the significant and persistent verbal WM disorders previously described. The results of Holmes et al. (80) only reported improved visuospatial skills in their participants. In a more recent study, Henry and colleagues (84) used a short and adaptative WM training, based on listening recall and odd one out span, in 47 children with DLD aged 6 to 10, randomly assigned into WM training or control training. Their results showed improvement in the children benefitting from the experimental training in WM itself as well as in global sentence comprehension [= subtest of the Assessment of Comprehension and Expression (85)], but not in receptive grammar [TROG-2 (86)]. Although very interesting, these results do not inform us about the effect of WM training on expressive syntax.

It is in this context that our team has developed a new WM training program, which is based on previous studies dealing with the predictive relationship between WM and complex syntax in children with TD and DLD (4, 9). As such, our program focuses on the specific WM aspects that have been shown to be predictive of syntactic performance, namely simple and complex verbal spans. Three studies have already been published on the results of this WM training, two in children with DLD (11, 12) and one in children with autism spectrum disorder (87). They clearly demonstrated the presence of (1) direct effects on WM itself, with better performance after training in verbal WM tasks; (2) transfer effects on syntax, with better performance on tasks assessing elicited production and repetition of complex sentences. More precisely, the authors (11) found improved production of 3rd person accusative clitics, a clinical marker of DLD in French, which were assessed in an elicitation task, in 26 children with DLD, aged 6 to 12, who had benefitted from WM training. Conversely, no significant progression was found for an age-matched control DLD group who had received an alternative training based on scholastic activities. Similarly, the same authors (12) used the same protocol with 52 children with DLD aged from 6 to 12 years: better performance was observed after WM training in a task of relative clause repetition. Improvements were visible on three measures: the percentage of correctly repeated syllables, the percentage of respected target structures (subject and object relatives), and the percentage of structures for which the correct degree of embedding was replicated. On the other hand, no progression was observed for simple sentences which were matched with complex sentences in length (14 syllables) but did not include any embedding. In line with these promising results, the current study deals with the effect of WM training on more ecological measures, namely, on the spontaneous utterances produced in narratives by children with DLD.

### The current study

Being part of a broader study investigating the effects of WM training on syntax in children with neurodevelopmental disorders (11, 12, 87), the current study aims to evaluate the impact of the WM training program *Magic Memory* (88) on the syntactic complexity of the spontaneous speech of French-speaking children with DLD. More specifically, we seek to determine whether improvement in WM *via* WM training, which has previously been proven to be effective in DLD (11, 12), leads to an increase in spontaneous productions of complex syntactic structures as well as a decrease in ungrammatical utterances. We also use previous data from TD children of the same age in order to compare their syntactic performance to that of children with DLD. The originality of this work lies in the fact that we analyzed children's performance through spontaneous language samples, more precisely in the context of storytelling, which is quite rare in the literature, as it is a very time-consuming procedure. As for our hypotheses, we predict that:

i. Syntactic related scores of the TD group will be, overall, significantly higher than those of the DLD group. This preliminary hypothesis would replicate the results already obtained in the literature [see (9) or (40)] and would validate the presence of complex syntax deficits in our specific population of participants with DLD.
ii. Participants with DLD trained in WM will increase in number of produced complex sentences in storytelling, by either producing a larger amount of such structures, or by producing more syntactically complex sentences. This main hypothesis could be reflected in several ways:
  - Using intragroup comparisons, post-test scores of children with DLD trained in WM should be higher than those obtained in pre-test, which should not be the case of age-matched control DLD children who followed an alternative, scholastic, training.
  - With intergroup comparisons, both groups of participants with DLD should have comparable performance in pre-test, but at post-test, the syntactic scores of those who were trained in WM should be higher than those in the control group.
  - Using direct comparison of gains (i.e., in measuring the difference between post-test and pre-test scores), we expect gains to be significantly higher in the WM trained group, compared to the control group.

## Method

### Participants

All our participants with DLD are part of the cohort of our previous studies which found WM improvement and transfer effects on expressive syntax after WM training (11, 12). The DLD group consisted of 39 French-speaking participants aged 6;0 to 12;2 at the beginning of the training (M_age _= 9;2; SD = 2;5), including 30 boys and 9 girls^4^, 35 monolinguals and 4 simultaneous bilinguals. Twenty of these participants were assigned to the WM training group “Magic Memory” (MM) and the 19 others^5^ to the scholastic (SQULA, Squla Inc. 2017) control training group. Participants were assigned to their group following a semi-randomized procedure considering the number of participants, their age and gender in order to obtain two comparable groups. Table 1 summarizes the main characteristics of each training group.

**Table 1:** Characteristics of training groups of participants with DLD.

DLD groups	N	Mean age (SD)	Age range	Gender	N bilinguals
DLD^MM^	20	9;7 (1;8)	6;7–12;5	F = 3; M = 17	3
DLD^SQULA^	19	9;4 (1;10)	6;0–11;9	F = 6; M = 13	1

^MM^ , Magic Memory (WM training group); ^SQULA^, scholastic (alternative training group).

The TD group consisted of 40 French-speaking monolingual participants aged 5;11 to 12;5 (M_age _= 9;2; SD = 2;2), including 23 boys and 17 girls. Spontaneous data of these participants came from the studies of Delage and Frauenfelder (4, 9). A Kruskal-Wallis test by rank, performed on the three groups (TD, DLD^MM^ and DLD^SQULA^), confirmed that there was not a significant effect of age (*p* = .9). Participants with DLD were recruited according to the following criteria: (i) their age must be between 6 and 12 years old, because this is the age where a predictive relationship has been demonstrated between complex syntax and WM scores in DLD (9); (ii) they must have been diagnosed with DLD by a qualified speech-language therapist; (iii) their WM and syntactic scores must be below average at the beginning of the training. For this purpose, we evaluated their expressive syntax with a common French standardized language test [BILO-3C (90)] and impairment was confirmed for all participants with scores of at least 1.25 standard deviation (SD) below age-specific norms. We also used a standardized assessment of verbal working memory in French (91), to assess WM for simple and complex spans. All participants with DLD displayed weak performance, with at least 1.25 SD below the normative mean on a minimum of three of the six WM tasks; (iv) their non-verbal reasoning scores had to be in the norm, with scores above the 10th percentile in a nonverbal reasoning task [Raven's Colored Progressive Matrices (92)], thereby excluding any risk of intellectual disability; (v) DLD had to be the only diagnosed disorder, with no known differentiating condition [as defined by the CATALISE group (7)]; (vi) all participants were required to be French-speaking monolinguals or simultaneous bilinguals, so that possible errors could not be attributed to L2 acquisition. Table 2 presents mean standard deviations (as compared to the norms of each task) of participants with DLD for standardized assessments, namely for expressive grammar, working memory (with composite scores for simple and complex spans) and non-verbal reasoning. The groups did not differ for any of these measures (all *p* > .2). As for TD participants, (i) their age had to be between 6 and 12 years old in order to match the DLD sample; (ii) they were required to have never been diagnosed with any language impairment and to have never received speech-language therapy; (iii) they needed to be French-speaking monolinguals or simultaneous bilinguals.

**Table 2:** Standardized assessments: non-verbal reasoning, expressive grammar and working memory.

DLD groups	Non-verbal reasoning (SD)	Expressive grammar (SD)	Working memory (SD)	
			Simple spans	Complex spans
DLD^MM^	−0.5 (0.6)	−3 (1.7)	−1.7 (1.1)	−2.0 (0.9)
DLD^SQULA^	−0.4 (0.8)	−3.2 (1.8)	−1.8 (0.8)	−2.5 (0.6)

SD, mean standard deviations (relative to the norms of each test); ^MM^, Magic Memory (WM training group); ^SQULA^, scholastic (alternative training group).

### Trainings

The alternative training program *SQULA* (https://www.squla.fr/) focuses on school-related skills and is designed to be used at home as an educational support tool. It is divided into several thematic categories such as mathematics, geography, or English as a foreign language. Given that these activities do not involve WM or syntactic training and offer school-based activities ranging from kindergarten to fifth grade (thus corresponding to our age range), they appeared particularly suitable as an alternative training. The activities are composed of recreational exercises on a digital medium, with access to performance feedback, thus resembling our target WM training.

Our experimental training program, *Magic Memory* “MM”, is described in detail in our previous studies (11, 12, 87). Compliance and progression of participants were directly recorded by the software^6^. It focuses on simple and complex verbal spans through five activities (lasting 5 min each), presented in the form of games (see Supplementary Appendix A). Both training programs (*SQULA* and MM) shared the same duration and format: for 8 weeks each participant completed three 30-minute training sessions per week, yielding a total of 12 hours per participant. Both programs were provided in computerized format, either on iPads or computers, depending on the material available at children's homes. Master students attended the first two training sessions of every participant to ensure that the provided instructions were followed, and they then attended training sessions at least once every two weeks to ensure the successful completion of the training. Participants were also promised a small reward, such as stickers, to motivate them to participate in the training. Approval for the research was obtained from the Ethics Committee of the University of Geneva. All parents received detailed information on the study and signed the consent form to approve the participation of their child.

### General procedure

For DLD participants, the overall procedure of testing included 11 tasks assessing WM capacities and various syntactic structures in production; these tasks were administered to participants one week before the trainings (i.e., **pretest**) and one week after the training (i.e., **posttest**). Our previous studies (11, 12) report the results of MM training on WM measures and on syntactic tasks, i.e., on tasks assessing elicited production of 3rd person accusative clitics and repetition of relative clauses. The task of interest for the current study, namely a storytelling task, was the ninth administered task within the overall testing procedure, which took place over two sessions. For this task, each participant was asked to tell a story based on a wordless picture book commonly used to elicit narratives (*The Frog Story* (93)). The participant's story was recorded only after each page from the book was first reviewed with the examiner to make sure the pictures were clearly understood by the participants who could ask for clarification at this point about the events, characters or items in the images. Then, the examiners recorded each story as an audio file. Examiners strictly followed the same experimental procedure with each participant, using specific protocol guidelines: they were asked to intervene as little as possible to avoid priming specific syntactic structures. Hence, they only reacted with neutral feedback, such as « mmh » or « okay », or used encouragements when the participant remained silent for too long (e.g., “Well done! Let's continue!”). The same picture book was used for the pretest and posttest. As for the TD group, the data came from previous studies (4, 9). Spontaneous language samples were elicited during a conversation between the child and an examiner who followed a defined script including a request for a narrative depending on the child's age (e.g.: *can you tell me about a movie or book you've seen/read recently? What was the story?*).

### Language measures in spontaneous language sample analysis

Table 3 presents the language measures we selected for our analyses of the language samples. The first measure, the mean length of utterances (MLU, calculated using words), is a well-known indicator of a child's ability to use complex language and is very often employed in studies on syntax in DLD (9, 15, 37, 40). Even if it is not a measure of complex syntax *per se*, longer utterances suggest more complex speech, as they inevitably require more syntactic operations for sentence formation, i.e., more external merges (43)^7^. Another important measure is the rate of embedding and of multiple embedding, also measured in Delage and Frauenfelder's studies (4, 9), which evaluates syntactic complexity in more detail by focusing on one's ability to use subordinate clauses. Multiple embedding in particular indicates a high degree of syntactic complexity (4, 37). The last measure we explored was the rate of erroneous complex utterances, which refers to the production of grammatical errors produced by children in such complex sentences. Such errors include the omission/substitution of any grammatical markers, such as complementizers, verbal inflection or gender marking, or even syntax errors related to word order.

**Table 3:** Language measures.

Mean length of utterances (MLU)	= average number of words per utterance from a participant's speech sample.
Rate of embedding (%)	= ratio of embedded clauses over the number of verbal utterances.
Rate of multiple embedding (%)	= ratio of embedded clauses that presents a degree of embedding strictly superior to 1 over the number of verbal utterances produced by the participant. A degree of embedding superior to 1 refers to a clause embedded within another embedded clause.
Rate of erroneous complex utterances (%)	= ratio of complex utterances that include an error over the total number of complex utterances. Only morphological or syntactic errors have been considered, not lexical or phonological ones.

Here is an example of a degree 2 of embedding provided by a participant with DLD (7;11), where [NF] refers to an infinitive predicate: “là il veut [PR] faire [SUB1] [NF] tomber [SUB2] [NF] la niche”. “There, he wants to make the doghouse fall”.

### Extraction of the data

For participants with DLD, a spoken corpus was created from the audio recordings, where one transcription corresponds to one story, *i.e.,* one testing session. This gave us a corpus of 78 stories, from which 40 (20 for pretest and 20 for posttest) belonged to the DLD^MM^ group and 38 (19 for pretest and 19 for posttest) belonged to the DLD^SQULA^ group. The mean length of the transcripts was 43.4 utterances for the DLD^MM^ group and 46.3 for the DLD^SQULA^ group, this difference not being significant (*p* = .8). Transcriptions were made by a master's student in linguistics who was a native French speaker. Furthermore, an expert linguist checked 20% of the transcriptions, with word-by-word reliability above 90%. Each transcription was systematically done following the same method, using the MacWhinney transcription guide, and analyzed with CLAN (95). As for the 40 samples of TD children, approximately 60 utterances per child had already been analyzed for use in previous studies. The studies conducted on these samples considered the same syntactic measures as those in our sample of DLD participants, using the same calculation methods. Transcript reliability was calculated for 10% of the TD transcripts with percentage agreement above 90% (4, 9). For both DLD and TD participants, utterances were divided by clausal units, with one utterance consisting of one main clause and its embedded clauses, following segmentation criteria defined by Rondal (96).

## Results

### Preliminary hypothesis: DLD vs. TD in syntactic performance

Table 4 shows the pretest scores from the DLD sample from both groups (MM and SQULA, *N* = 39) as well as those of the TD group (*N* = 40). As expected, the results confirmed a clear difference between the two groups, with TD outperforming DLD children, on all the language measures. The difference with the larger effect size (Cohen's *d* = 1) corresponds to the rate of embedding, i.e., the proportion of complex utterances produced by participants over the total number of their utterances. In sum, children with DLD produced shorter utterances than TDs, with fewer embedded causes (notably almost no multiple embedding), and more errors in complex utterances. Examples (c) and (d) illustrate erroneous complex sentences, with multiple embedding for (d), produced by participants with DLD from our study.

(c) DLD (7;11): il a essayé de manger [PR] le miel qui [*] préparait [SUB1] les abeilles.^8^ “he tried to eat the honey that prepared the bees’.
  → Target in French: il a essayé de manger le miel que préparaient les abeilles.
  → Target in English: he tried to eat the honey that the bees were preparing.
(d) DLD (8;6): c' était [PR] l' hibou qui lui [*] avait fait [SUB1] tomber [SUB2] [NF] [CAU] de l' arbre. “it was the owl that made to him fall from the tree”
  → Target in French: c'était le hibou qui l'avait fait tomber de l'arbre.
  → Target in English: it was the owl that made him fall from the tree.

**Table 4:** TD versus DLD: measures of complex syntax.

Language measures	M_TD_ (SD)	M_DLD_ (SD)	TD > DLD
MLU	7.7 (1.1)	6.1 (1.2)	*t* (77) = −6.0, *p* < .001 Cohen's d = 0.7
Rate of embedding / *N* verbal utterances	36.8% (13)	13.1% (9.6)	*t* (77) = −9.2, *p* < .001 Cohen's d = 1.0
Rate of multiple embedding / *N* verbal utterances	6.6% (6.1)	0.9% (1.8)	*t* (77) = −5.6, *p *< .001 Cohen's d = 0.6
Rate of erroneous complex utterances / *N* complex utterances	9.3% (7.1)	31.9% (28)	*t* (77) = 4.9, *p* < .001 Cohen's *d* = 0.5

MLU, mean length of utterances.

### Main hypothesis: Effect of training

As for our main hypothesis, we predicted that participants with DLD who were trained in WM (DLD^MM^) would increase the syntactic complexity they used in their narratives in posttest, as opposed to participants with DLD who followed the alternative training (DLD^SQULA^). Statistical analyses conducted to test this hypothesis have been conducted with non-parametric tests due to the small number of DLD participants in each group (20 DLD^MM^, 19 DLD^SQULA^).

#### Intragroup comparisons

Table 5 compares the pretest scores and the posttest scores for each language measure in both groups, using the Wilcoxon test for intragroup comparisons. No measure showed significant progression between pre- and posttest, but we observe a decrease in the performance of the control group (DLD^SQULA^) for the rate of embedding. At the same time, and although the difference is not significant (*p* = 0.1), the DLD^MM^ group improved on this measure from a rate of 12.7% in pretest to a rate of 15.9% in posttest. Finally, the rate of erroneous complex utterances appears to be slightly higher in posttest, as compared to pretest, for both groups.

**Table 5:** Pre- and post-training scores in DLD^MM^ and DLD^SQULA^.

Language measures	DLD^MM^				DLD^SQULA^			
	M (SD) pretest	M (SD) posttest	Z	*p-value*	M (SD) pretest	M (SD) posttest	Z	*p-value*
MLU	6.1 (1.5)	6.1 (1)	0.34	.7 ns	6.0 (0.9)	5.9 (0.9)	0.28	.8 ns
Rate of embedding	12.7% (9.7)	15.9% (9)	1.57	.1 ns	13.6% (9.6)	9.9% (8.7)	2.34	.019*
Rate of multiple embedding	0.8% (1.4)	1.1% (1.5)	0.53	.6 ns	1.0% (2.2)	0.4% (1)	1.48	.1 ns
Rate of erroneous complex utterances	24.7% (25.5)	32.8% (22.6)	0.75	.4 ns	38.6% (29.3)	46.5% (29.8)	0.22	.8 ns

^MM^ , Magic Memory (WM training group); ^SQULA^, scholastic (alternative training group).

* indicates a significant difference.

#### Intergroup comparisons

Mann-Whitney tests used for intergroup comparisons, namely DLD^MM^ vs. DLD^SQULA^, confirmed that the performance of both groups did not differ at pretest, for all the measures: MLU (*p* = .9), rate of embedding (*p* = .6), rate of multiple embedding (*p* = .8) and rate of erroneous complex utterances (*p* = .1). As for the posttest scores however, the two groups significantly differed for the rate of embedding (DLD^MM^: 15.9% > DLD^SQULA^: 9.9%, U = 111, *p* = .026). The other measures did not distinguish the two groups: MLU (*p* = .7), rate of multiple embedding (*p* = .2), and rate of erroneous complex utterances (*p* = .2).

#### Comparison of gains

Since previous results revealed different patterns of performance between DLD^MM^ and DLD^SQULA^ at post-test, as opposed to pretest, we calculated the difference between posttest and pretest scores for each language measure. Table 6 compares these “measures of gains” between both training groups; it appears that, as expected, the DLD^MM^ group outperformed the DLD^SQULA^ group for two measures of gains, namely those of rate of embedding and of multiple embedding.

**Table 6:** Gains (between post-training and pre-training scores) in DLD^MM^ and DLD^SQULA^.

Gains in language measures	M_DLD_^MM^ (SD)	M_DLD_^SQULA^ (SD)	DLD^MM ^> DLD^SQULA^
MLU	−0.03 (1.1)	−0.08 (0.7)	U = − 0.2, *p* = .8 ns
Rate of embedding	+3.22% (9.7)	−3.73% (6.9)	U = 97, *p* = .009 *r* = 0,4
Rate of multiple embedding	+2.64% (9.4)	−0.61% (1.8)	U = 118, *p* = .038 *r* = 0,3
Rate of erroneous complex utterances	+11.38% (24.1)	+4.61% (28.6)	U = 136, *p* = .1 ns

^MM^ , Magic Memory (WM training group); ^SQULA^, scholastic (alternative training group).

Although our samples were not large enough to use parametrical analyses, and therefore to have enough statistical power, we conducted an exploratory analysis using repeated measures ANOVAs with time (pretest, posttest) as the within subject variable and training type (^MM,^ ^SQULA^) as the between subject factor. Results are to be considered with extreme caution for the above-mentioned reasons, but they seem to confirm a treatment effect with a statistically significant interaction effect of time by type of training type for the rate of embedding [F(1, 37) = 6.31, *p *= .016, *η*2 = .14]. Note, however, that the effect size is small and that no such interaction was observed for multiple embedding (*p* = 0.1).

#### Further analyses

As for further exploration of our data, we investigated several possible links between our clinical data (age and gender) and language measures. It appears that, considering the entire group of DLD participants, age correlated significantly with MLU at both pre- and posttest (respectively *r*_s_ = 0.35, *p* = .03, *r*_s_ = 0.33, *p* = .04). No other language measure showed such correlation with age^9^. As for sex, we compared the different language measures of the 30 boys and the 9 girls and did not find any differences (all *p* > .2). Lastly, we focused on the measure of erroneous complex utterances, which showed unexpected results, since there was a tendency for all DLD participants to produce more errors in posttest, as compared to pretest. This increase in the rate of erroneous complex utterances did not correlate with other measures of gains in language measures if we consider the total group of DLD participants (*N* = 39, all *p* > .2). However, if we consider the two groups separately (DLD^MM^, DLD^SQULA^), the improvement in erroneous complex utterances in the DLD^MM^ group showed a marginal tendency, with gain in the rates of embedding (*r*_s_ = 0.44 *p* = .054) and of multiple embedding (*r*_s_ = 0.43, *p* = .06). Such tendential correlations were absent on the DLD^SQULA^ group (all *p* > .2). In other words, it seems that an increase in the use of embedding goes in line with an increase in the errors produced in such complex utterances.

## Discussion

Our study aimed to assess the impact of our WM training program on the syntactic complexity of the spontaneous speech of French-speaking children with DLD. Syntactic complexity in narrative samples of 20 children with DLD aged 6 to 12 who had been trained in WM was analyzed (1) *via* intragroup comparisons before and after training, and (2) *via* intergroup comparisons with 19 age-matched children with DLD who had followed an alternative, control training. Syntactic performance of the total DLD sample (*N* = 39) was also compared to that of 40 TD children of the same age. Global results confirmed the severity of the syntactic impairment in children with DLD, as opposed to TD children. Findings may also suggest an increase in the use of embedded clauses in children who participated in the WM training, whereas such progression was absent in the DLD control group. However, the latter results should be viewed with caution, given the lack of statistical power due to the small sample size.

### Syntactic differences between TD and DLD

Differences in syntactic performance between TD and DLD children support previous spontaneous speech findings (9, 38, 40, 42, 97). Although all measures showed strong significant differences between DLD and TD, the largest difference, i.e., with the largest effect size, was found for the rate of embedding, whose effect size was double that obtained for the rate of erroneous complex utterances. This result highlights DLD children's vulnerability producing complex sentences, which is one of the specific characteristics of the disorder. This also makes it possible to orient diagnosis in situations where the identification of DLD is challenging, as is the case for DLD in bilingual children (98, 99). On this topic, Scheidnes and Tuller (36) found that the frequency of embedded clauses produced in spontaneous language by children with DLD was lower than that of sequential bilingual TD children^10^, whereas both groups displayed similar rates of morphosyntactic errors. Hence, rate of embedding measured in spontaneous speech could provide clues for differentiating “true” DLD from transient language difficulties due to lack of exposure to the L2. Unfortunately, syntactic analysis of spontaneous language samples is almost never performed by speech-language therapists, due to the time-consuming nature of the task as well as clinicians' self-proclaimed lack of knowledge and skills evaluating spontaneous speech data (100). However, embedding can be assessed *via* sentence repetition tasks that control precisely for this aspect, by manipulating the type and the number of embedded clauses, such as the Language Impairment Testing in Multilingual Settings (LITMUS) sentence repetition (SR) task, which was created to diagnose DLD in bilingual children (101).

The empirical results reported in this work should nonetheless be considered in light of some methodological shortcomings. For example, the conditions for collecting spontaneous language samples differed between the DLD and TD groups of children: DLD participants were asked to tell a story based on a single picture book, whereas language samples of TD children consisted of less formal interviews during which the participants were asked to tell a story based on a book or movie they had enjoyed. Depending on the participant's style, some narratives were very detailed while others produced little narrative, preferring a more spontaneous conversation, on leisure activities for example. However, it has been shown in the literature that spontaneous language samples obtained during storytelling, i.e., in a more directed context, contain higher MLUs in TD children, compared to more natural interaction situations, either conversation or free play (102–104), possibly related to the increasingly frequent exposure to written language (105). Kover et al. (106) also reported higher MLUs in narrative contexts than in conversation in adolescents with Fragile X or Down syndromes. In this sense, the DLD children in our study would be at an advantage compared to the TD children who were recorded in a less structured situation. It should nevertheless be noted that some authors, such as Wetherell et al. (107), have pointed out that adolescents with DLD demonstrate even greater syntactic difficulties in storytelling than in conversational contexts, in which they are able to manage their difficulties more discreetly. Thus, it remains unclear whether the different language collection conditions of the two groups may have influenced our results.

### Effect of WM training on complex syntax

As for the effect of WM training, our intragroup comparisons did not show any significant progression between the pre- and post-training phases. At the qualitative level, however, an increase was observed for the WM-trained group (DLD^MM^) for the rate of embedding, which contrasts with the significant unexpected decrease observed for this same measure for the DLD^SQULA^ control group. Despite these differences in performance, the lack of significant progress in the DLD^MM^ children is inconsistent with our previous results showing significant improvement, after training, in the production of accusative clitics (11) as well as in repetition of complex sentences (12). It should nevertheless be noted that these last two tasks are particularly well suited for monitoring grammatical progress, since the participants have no choice in the production of their structures. For instance, in the sentence repetition task, they must repeat a subject or object relative in a structure with one or two degrees of embedding, and if they fail to do so, they get a score of zero. In our narrative elicitation task, even though the story is the same for all participants, the participants are free to choose the structures that are most spontaneous/natural to them and, therefore unconsciously, those that cause them the least problem in terms of their grammatical complexity. This brings to mind the idea of compensation evoked by Hamann et al. (40) and Tuller et al. (37) who reported that, in spontaneous language samples, adolescents with DLD tended to produce less ungrammatical utterances than younger participants with the same condition by avoiding more embedded structures. To do this, they tended to use compensatory strategies such as the juxtaposition of two simple sentences or the conjunction of two simple sentences, instead of complex sentences like relatives.

In any case, even if our direct intragroup comparisons are not statistically compelling, our intergroup analyses still seem to show some improvement in the use of embedded sentences in the trained group (DLD^MM^). While the two training groups did not differ at pretest for any of the syntactic measures^11^, they significantly differed at posttest for the rate of embedding, with rates being higher for DLD^MM^ children than for children in the control DLD^SQULA^ group. When we looked more closely at the results and compared the gains of both groups (calculated from the difference between posttest and pretest scores), such gains were higher for the DLD^MM^ group for the two measures closely linked to sentential embedding, namely rate of embedding and of multiple embedding. This difference in gains was confirmed by an exploratory analysis, using repeated measures, which found a time by training group interaction for the rate of embedding. Unquestionably, these results should be replicated in subsequent studies that include more participants so as to more solidly conclude that working memory training can indeed be efficient for embedding, especially because the observed difference between the trained and control groups at posttest was dependent on the worsening of the control group's performance, a point we discuss in section “Unexpected results”. However, these finding for the DLD^MM^ group are nevertheless encouraging since they are in line with previous results showing improvement in the ability to respect the expected degree of embedding in repetition of complex sentences after WM training (12).

To recall, Delage and Frauenfelder (9) showed that the rate of embedding measured in children with DLD in their study (*N* = 28, aged 6–12) was significantly predicted by their WM abilities, especially with respect to their performance on verbal simple span tasks. This discovered fueled the format of our five WM training activities, which indeed, all target verbal simple spans. It would thus seem that improvement in WM performance measured by simple spans, already confirmed by our previous studies on the same cohort (11, 12), supports the mastery of complex linguistic structures, not only in repetition (12), but also, apparently, in spontaneous language production as suggested in the present study. Finally, this improvement, if it did exist, cannot be explained by the fact that the participants had already told the same story 10 weeks earlier, i.e., before training, because the control participants with DLD showed no improvement on these same measures. This result seems to suggest that the progress we observed can be attributed to the WM training program *Magic Memory*, and not to a training effect in general, nor to a test-retest effect.

### Unexpected results

It was expected that MLU, a base measure of complexity of language (37), would follow the same progression as the rate of embedded clauses, since the complexification of the children's productions would logically entail more words to produce complex structures, such as the use of complementizers and of predicates in embedded clauses. Previous studies have reported a strong positive correlation between MLU and clausal density in natural discourse (108, 109). However, we observed a stagnation of this measure in posttest, as compared to pretest, in both groups, including the DLD^MM^ one. This result might be explained by the fact that participants were asked to tell the same story at pre- and posttest. As such, it is possible that they would naturally shorten their story in the posttest phase, by better synthesizing the narrative elements of the story.

Moreover, we did not expect to find a significant decrease in rate of embedding by the DLD^SQULA^ group. Since it seems unlikely that children's complex syntax ability worsened, we can imagine that they were less interested in recalling the same story as in the pretest, and thus used simpler sentences without details that could be added by using complex syntactic structures^12^. On the other hand, DLD^MM^ children were in the same situation, yet they used more complex sentences, even if it did not reach significance. It is possible that the freeing up cognitive resources, linked to the cognitive training they underwent, facilitated the use of complex syntactic structures, despite the redundancy of the two narratives. As possible bias, a placebo effect cannot explain these results because the children of both groups were unaware of the final objective of the study (i.e., that we wished to see an improvement of their syntactic capacities). A Rosenthal effect also seems unlikely in the sense that the experimenter intervened minimally in the children's narrative.

Additionally, our further analyses focused on the evolution of erroneous complex utterances, which tended to be higher in post-test in both training groups. If we consider the marginal correlations we obtained in DLD^MM^ between the gain in the use of embedding and the increase in erroneous complex utterances, it appears that an increase in grammatical errors could be linked to the complexification of the utterances produced by the children in our study. Since embedding is a key component of computational complexity in the DCH approach (30), it is logical to observe that complex utterances contained more morphosyntactic errors than utterances containing no embedded clause [see also (37)]. In other words, a larger number of produced embedded utterances would go hand in hand with an increase in grammatical errors. Several questions therefore arise: is it better to promote syntactic complexity in these still young children, with WM training programs such as ours, possibly combined with explicit grammar learning strategies that have already proved effective in English-speaking DLD children and adolescents (110–112)? Does this reinforcement of syntactic complexity necessarily come at the cost of decreased syntactic accuracy, when an abundance of grammatical errors could be stigmatizing in everyday life? Or should we consider that less complex but less erroneous language, would promote better social integration, as suggested by Tuller et al. (37) on the basis of results of French-speaking adolescents with DLD? These questions remain open and are beyond the scope of this work.

### Limitations

The first limitation concerns the small number of participants that should be increased for future studies. We also acknowledge that for some language measures, data were limited. For example, there were only 15 occurrences of multiple embedding for all participants in the DLD^MM^ group at posttest. However, this low rate of deep embedding is also indicative of DLD children's difficulties with complex syntax. For example, Delage and Frauenfelder (9) and Tuller et al. (37) also reported very low rates on this measure for children and adolescents. Next, we only considered the phenomena of embedding in this study, while the training could have had more beneficial effects for other syntactic structures in the DLD population. In particular, with the aim of obtaining more robust results, we could have focused on structures involving syntactic movement, and thus a non-canonical order of sentence constituents, such as accusative clitics or object relatives, since our previous studies have identified a positive effect of WM training specifically for these structures (11, 12). To do so, one could imagine inducing semi-spontaneous speech to maintain the benefits of more ecological data, while having access to more occurrences of the targeted syntactic structures. This could be achieved by slightly modifying the narrative task we presented in this study, to a question oriented narrative task, where the participants are still speaking freely but with questions to induce the production of the targeted structures. Such questions could be: “And there, what is he doing with the frog?” in order to elicit accusative clitics as in “il **la** cherche” “he is looking for it”. Finally, in the absence of follow-up testing, it is not possible to know the long-term effects of our intervention on measures of spontaneous language. However, in our study conducted with children with autistic spectrum disorder (87), we were able to re-test 26 of the 30 trained children, aged 6 to 12, and the improvement obtained in the repetition of complex sentences was maintained three months after training. A future study should therefore be able to investigate whether the modest effects we observe in spontaneous speech with a population of DLD children follow the same trend.

### Clinical implications

We were able to confirm the presence of syntactic deficits in our population of children with DLD *via* an analysis of spontaneous speech samples. Such an approach is invaluable as it contributes to the evaluation of syntactic skills in a naturalistic context, as opposed to common standardized tasks. Clinicians should be encouraged and trained to conduct such language sample analyses in order to gain a more ecological view of their patients' performance in a situation that closely resembles their daily interactions. In a recent scientific conference targeting speech-language therapist, Zwitzerlood and Klatte (113) further argued that this type of analysis constitutes the “gold standard” for in-depth diagnosis and goal setting for productive morphosyntax in children with DLD. As for trends concerning the positive effect of our WM training, they are consistent with the positive findings of our previous studies on more experimental tasks and encourage speech-language pathologists to use verbal WM training with their patients who have limitations in this area to obtain a positive transfer effect on syntax. Recall that such training studies conducted with children with language disorders are still rare in the literature, apart from ones by Holmes et al. (80) and Henry et al. (84). We hope that the present work will open the door for future WM training studies aimed at children with DLD.

## Conclusion

The present research, combined with our previous studies using the same WM training (11, 12), supports the existing literature about deficits in complex syntax in children with DLD, while offering promising trends in the direction of transfer effects of verbal WM training on the capacity of participants with DLD to produce embedded clauses. Even if the results of the present study only suggest, but cannot prove, an increase in the use of embedding which could be attributed to our new WM training program, *Magic Memory*, they underline the importance of investigating transfer effects to spontaneous speech, despite the fact that it is more difficult to show evidence of improvement in these more ecologically valid tasks, compared to (artificial) experimental tasks. Indeed, the fact that our data came from spontaneous speech is a valuable aspect of this study, contributing to an ecological view of syntactic skills in DLD, and our preliminary results warrant follow-up investigations of the trends observed in the rate of embedding in natural speech.

## Data Availability

The raw data supporting the conclusions of this article will be made available by the authors, without undue reservation.

## Footnotes

^1^“It could be that unbounded Merge, and whatever else is involved in UG (= Universal Grammar), is present at once, but only manifested in limited ways for extraneous reasons (memory and attention limitations and the like)” [(44), p. 12]. Note that this approach contrasts with the view whereby child grammar is thought to be in an immature of incomplete state [see (45)].

^2^See (53), as well as the revised version (1).

^3^Note that these relationships have also been reported for other neurodevelopmental disorders, such as autism spectrum disorder (59) or specific learning disorder (75).

^4^This sex ratio is close to the clinical prevalence rate of DLD, where males are twice as likely to be affected than females (89).

^5^One child, initially included in the SQULA group, is missing because the audio recordings could not be transcribed, due to the poor quality of the file.

^6^For more information, see Stanford et al. (11), who reported the significant progression of participants throughout the 24 sessions on the WM tasks that had been directly trained.

^7^See also Brown (94, p. 53–54): “*The MLU is an excellent simple index of grammatical development because almost every new kind of knowledge increases length: the number of semantic roles expressed in a sentence, the addition of obligatory morphemes, coding modulation of meaning […] and, of course, embedding and coordinating”.*

^8^PR, main clause; SUB, subordinate clause; NF, non-finite clause; CAU, causative. Numbers indicate the level of embedding of the subordinate clause. [*] follows a grammatical error.

^9^Note that this absence of age progression in the use of complex utterances in children with DLD justifies the wide range of ages of our participants (6–12 years old).

^10^Note that we cannot proceed to analyses in distinguishing performance of mono- and bilinguals in the present study since all TD children were monolinguals, and only four children with DLD were simultaneous bilinguals, sequential bilingualism being an exclusionary factor.

^11^This result was crucial to the study as it is the prerequisite to draw any conclusion about the effectiveness of our WM training program.

^12^Note that this is not a phenomenon that is limited to a few participants. When examining the individual results, 13 participants (out of 19) in the DLD^SQULA^ group have a negative gain on this measure, compared to only 4 (out of 20) in the DLD^MM^ group.
