# Supplementary material for: Working memory training in children with developmental language disorder: Effects on complex syntax in narratives
Source: Front Rehabil Sci. 2023 Jan 4;3:1068959. doi: 10.3389/fresc.2022.1068959 (PMC9846049; doi:10.3389/fresc.2022.1068959)
Supplement: Supplementary file 1 [file Datasheet1.docx]

**Appendix A. Working memory training: *Magic Memory* activities.**

*Activity 1: Serial order memory*

The first activity required participants to store a list of verbal items in successive order, with the aim of training serial order short-term memory capacity. In this activity, inspired by Majerus and colleagues (2006), participants heard a series of familiar monosyllabic words and then had to place images corresponding to the words in the correct order, i.e. the order in which the words had been presented. The number of presented items increased as the participant’s performance improved, thus adapting the complexity of the task to the child’s ability. Specifically, there is an increase of one item after two successful attempts at the same span level and a decrease of one item after two unsuccessful attempts at the same span level.

*Activity 2: WM updating*

We created an adapted version of the classic n-back task in order to train WM updating with the second activity. While doing this activity, the participants saw a sequence of visual stimuli and were told to indicate when the current stimulus matched the one from n steps earlier in the sequence: 1-back, 2-back or 3-back according to the level of difficulty. To make the activity more or less challenging for the participant, the load number adjusted automatically to the ability of the participant based on his or her performance on the previous trials. More precisely, when the participant achieved a score greater than 71% at the end of a session, the n-back level increased by one for the subsequent session; for scores below or equal to 50%, the n-back level decreased by one for the next session.

*Activity 3: Serial order and complex WM*

With the third activity, which aimed to train complex span, participants were required to store the order of familiar auditory stimuli (e.g. a ringing telephone, a crying baby or a barking dog) while simultaneously performing a secondary task, a visual comparison of quantity task. After having listened to a certain number of stimuli and completed a matching number of quantity comparison tasks, the participant was asked to place images corresponding to the sounds in their order of presentation. Based on the child’s performance, the number of presented stimuli increased or decreased throughout the task, on the same basis as in activity 1.

*Activities 4 and 5: Simple and complex span*

The fourth and fifth activities, inspired by the classic digit span task, were designed to train simple and complex WM span. Participants were instructed to retain the order of a series of color names (e.g. blue, red, green) and, for the fourth activity, participants were told to indicate the colors they had heard in order of presentation. The fifth activity followed the same procedure, but this time, the participants recalled the series of colors in reverse order. As with the other activities, the level of difficulty was adjusted to match the participant’s level, on the same basis as in activity 1.
